# Efficacy and safety of botulinum toxin a injection into urethral sphincter for underactive bladder

**DOI:** 10.1186/s12894-019-0490-4

**Published:** 2019-07-05

**Authors:** Guoqing Chen, Limin Liao, Fei Zhang

**Affiliations:** 1 Department of Urology, China Rehabilitation Research Center, Beijing, 100068 China; 2 Department of Urology, Capital Medical University, Beijing, China; 3 Department of Urology, Baotou Central Hospital, Baotou, 014040 China

**Keywords:** Urethral sphincter, Botulinum toxin type A, Underactive bladder, Residual urine volume

## Abstract

**Background:**

The aim of this retrospective study was to evaluate the clinical efficacy and safety of botulinum toxin type A (BTX-A) injection into the urethral sphincter to treat patients with underactive bladder (UAB).

**Methods:**

From September 2012 to December 2018, 35 patients with UAB who presented with dysuria were treated with BTX-A (Prosigne®, Lanzhou Biological Products, Lanzhou, China). All patients were evaluated using the International Continence Society standard for video-urodynamic examination before and 1 month after treatment. The index includes maximum urinary flow rate, detrusor leak point pressure, and maximum urethral pressure. Post-voiding residual urine volume was measured using ultrasound before, one and 3 months post injection.

**Results:**

After 1 month of treatment, the maximum flow rate increased from 2.5 ± 1.1 ml/s to 6.6 ± 1.7 ml/s (*P* < 0. 05). The maximum urethral pressure decreased from 73.5 ± 5.8 cmH_2_o to 45.6 ± 4.3cmH_2_O (*P* < 0. 05). The detrusor leak point pressure decreased from 69.9 ± 20.7cmH_2_O to 26.3 ± 7.4cmH_2_O (*P* < 0. 01). Post-voiding residual urine decreased from 282.8 ± 134.2 ml to 125.0 ± 92.1 ml (*P* < 0. 01) but increased to 270.1 ± 129.0 ml 3 months post injection. Of the 35 patients, 57.1% (20/35) relied on clean intermittent catheterization (CIC) before injection, but 75.0% (15/20) of them could partly void 1 month after injection, and 25%(5/20) could void without CIC. Eight patients showed hydronephrosis before treatment; in three of them, hydronephrosis decreased slightly, while it resolved in two. All patients were followed for three to 6 months, and the effect lasted for about two to 3 months. No serious adverse events occurred in any patient.

**Conclusions:**

The results suggest that Prosigne® injection into the urethral sphincter is an effective, safe, and inexpensive way to treat UAB.

## Background

Botulinum toxins (BTX) which derive from the Gram-positive coccus *Clostridium botulinum* are the most potent known naturally occurring neurotoxins [1]. They can paralyze striated muscle by blocking acetylcholine release at the presynapse. U.S. Food and Drug Administration approved BTX for the treatment of strabismus, blepharospasm, and hemifacial spasm in 1989 [2]. BTX can be classified into seven different types: A, B, C, D, E, F, and G according to its different immune antigens [3]. The first licensed serotype in clinical use was BTX-A with the trade name Botox® (Allergan Pharmaceuticals, Irvine, CA), but other brands also exist, including Dysport® (Ipsen Biopharm Ltd., Slough, UK), Xeomin® (Merz Pharmaceuticals UK Ltd., Hertfordshire, UK), Prosigne® (Lanzhou Biological Products, Lanzhou, China), and PurTox® (Mentor Corporation, Madison, WI) [4].

In recent years, BTX-A injection has been widely used in the treatment and research of lower urinary tract dysfunction [5]. The site of BTX-A injection is classified into simple detrusor injection for detrusor overactivity, simple sphincter injection, or detrusor-sphincter combined injection for sphincter spasm or detrusor sphincter dysfunction. Our department is the first in China to use BTX-A injection to treat lower urinary tract dysfunction. We used BTX-A (Prosigne®) injected into the detrusor for neurogenic detrusor overactivity [6] and interstitial cystitis [7] and achieved satisfactory results. We also used Prosigne® to inject into the urethral sphincter to treat underactive bladder (UAB). This retrospective study was to evaluate the efficacy and safety of Prosigne® in patients with UAB.

## Methods

All subjects signed their informed consent for inclusion before they participated in the study. The study was conducted in accordance with the Declaration of Helsinki, and the protocol was approved by the Ethics Committee of China Rehabilitation Research Center (Project identification code: 2018–053-1). Before treatment, all patients underwent video-urodynamic examination according to the International Continence Society standard [8], and the maximum urinary flow rate, maximum urethral pressure, and detrusor leak point pressure were recorded. According to the classification of urodynamic diagnosis, 26 patients had detrusor underactivity, and nine had acontractile detrusor. There was no bladder-ureter reflux, and urinary ultrasound showed that eight patients had mild hydronephrosis. Twenty of the 35 patients had previously relied on clean intermittent catheterization (CIC). The above indicators were reviewed 1 month after treatment. Residual urine volume was measured by ultrasound before, one and 3 months post injection, and the mean of three measurements was taken.

BTX-A (Prosigne®) was used in treatment. The patients were treated in the lithotomy position with routine insertion of a 21F cystourethroscope. After identifying the circular external sphincter under the endoscope, a 6F bladder injection needle was inserted into the external sphincter using cystourethroscopy. One-hundred units of BTX-A were diluted with 8 ml saline and injected into the external sphincter. In the direction of the 3, 6, 9, and 12 points of the external sphincter, two needles were injected longitudinally near each point, a total of 8 needles. Each injection was 1 ml and 1 or 2 cm deep at the latent injection site. And after the operation, Foley catheter was indwelled.

Using GraphPad Prism 8 software, all measurement data were expressed by mean + standard deviation; the paired t test and one-way ANOVA was used for group measurement data. *P* < 0.05 was considered to be statistically significant.

## Results

From September 2012 to December 2018, 35 patients with UAB and dysuria underwent BTX-A (Prosigne®) injection into their urethral sphincter in our hospital, including 21 men and 14 women aged 19 to 77 (41.85 + 15.80) years. Twenty-four patients had neurogenic bladder, and 11 had non-neurogenic, non-obstructive urinary retention. Specific etiologies are shown in Table 1. All patients were given a thorough explanation of the treatment and written informed consent before the injection.

**Table 1:** Patient’s etiologies

Etiology	Number of patients
Neurogenic decease	24
Cone horsetail injury	10
Spina bifida and meningocele	8
Spinal surgery	6
Non-neurogenic, non-obstructive urinary retention	11
Total	35

**Fig. 1:**
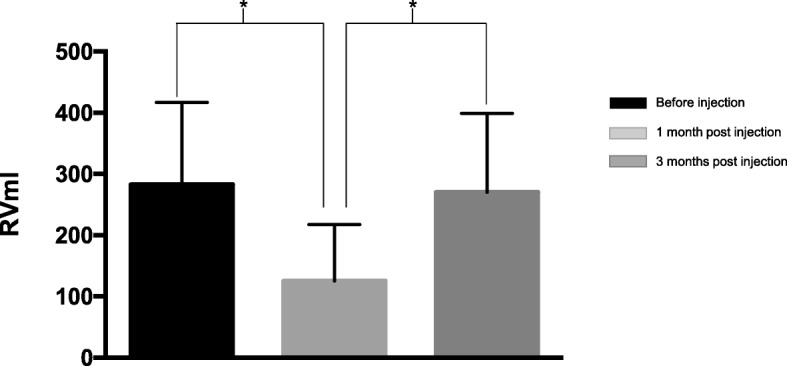
Residual urine volume before, one and three months post injection. *: *P* < 0.05

The catheter was removed 7 days after injection with BTX-A. One month after treatment, all patients underwent video-urodynamic examination. The maximum urinary flow rate increased (*P* < 0.05), while the maximum urethral pressure and detrusor leak point pressure reduced by statistically significant amounts (*P* < 0.05) (Table 2). Residual urine decreased significantly 1 month post injection (*P* < 0.05) but increased to the same level as before treatment 3 months post injection (Fig. 1). Of the 35 patients, 57.1% (20/35) relied on CIC before injection, but 75.0% (15/20) of them could partly void 1 month after injection, and 25% (5/20) could void without CIC.

**Table 2:** Comparison of urodynamic parameters in patients with underactive bladder before and after treatment

Urodynamic parameters	Baseline	One month
Pura.max (cmH_2_O)	7 73.5 ± 5.8	45.6 ± 4.3*
DLPP (cmH_2_O)	7 69.9 ± 20.7	26.3 ± 7.4*
Qmax (ml/s)	2. 2.5 ± 1.1	6.6 ± 1.7*

*DLPP* Detrusor leak point pressure, *Pura.max* Maximum urethral pressure, *Qmax* Maximum urinary flow rate. *:*p* < 0.05

Urinary ultrasonography was conducted 1 month after treatment. Three patients had hydronephrosis without obvious relief, three had slight relief from hydronephrosis, and two had resolution of their hydronephrosis. The follow-up period was three to 6 months, and the relief lasted two to 3 months. No serious adverse reactions occurred in any patient. None of the patients had incontinence before the procedure, but mild urinary stress incontinence occurred in five patients after treatment and resolved two or 3 months later.

## Discussion

UAB is a complicated clinical syndrome characterized by prolonged urination, with or without a sensation of incomplete bladder emptying, usually with hesitancy, a slow stream, and reduced sensation on filling [9]. According to possible mechanisms, UAB can be classified into three types: idiopathic, neurogenic, or myogenic [10]. In our study, 24 patients had neurogenic bladder, and 11 had non-neurogenic, non-obstructive urinary retention. Based on urodynamic examination, UAB can be classified into detrusor underactivity or acontractile detrusor [10]. In this study, 26 patients had detrusor underactivity, and nine had contractile detrusor.

Whether UAB was neurogenic or nonneurogenic, the main symptom of all patients was dysuria, which negatively affects quality of life-especially in patients with hydronephrosis due to ureteral reflux, in whom renal dysfunction may occur at any time and be life-threatening. Reducing residual urine, avoiding overdistension, and preventing upper urinary tract damage are considered appropriate management for patients with UAB [9].

For these patients, the gold standard of treatment is intermittent catheterization [10]. However, some young patients do not accept intermittent catheterization but try to excrete urine through manual-assisted urination. Because manual-assisted urination (Crede and Valsalva urination) may cause bladder pressure to exceed the safe range, this method could induce or aggravate upper urinary tract damage and is not recommended [11]. For some patients in stable condition with no upper urinary tract damage and a strong willingness to urinate autonomously, BTX-A injection into the urethral sphincter combined with autonomous urination can be considered. However, long-term follow-up is necessary during the application period.

BTX-A is a pathogenic substance and the most powerful natural neurotoxin in nature. This neurotoxin infiltrates the nerve endings of presynaptic cholinergic neurons, enters neurons through receptor-mediated endocytosis, and catalyzes the decomposition of SNAP-25 protein, promoting the fusion of synaptic vesicles. This cleavage inhibits the secretion of acetylcholine, resulting in temporary chemical denervation and loss or weakening of nerve activity in target organs [3]. Usually, the chemical denervation is reversible. Dykstra et al. first injected 100 units of BTX-A into the external urethral sphincter to treat patients with spinal cord injury in 1988 [12]. They concluded that the pressure of the urethra and bladder was reduced at the same time.

In this study, 35 patients with UAB underwent BTX-A injection into the urethral sphincter in our hospital. After treatment, the maximum urinary flow rate increased, while residual urine, maximum urethral pressure, and detrusor leak point pressure decreased.

The decrease of maximum urethral pressure and DLPP can effectively alleviate the effect of high bladder pressure on upper urinary tract. In this study, of eight patients with mild hydronephrosis before treatment, three had slight relief and two had resolution 1 month after injection. Fifteen patients still did not completely detach from CIC, but they can partially urinate autonomously and the frequency of CIC was reduced which improved the quality of life.

Although urethral BTX-A injection may increase the risk of urinary incontinence, this side effect gradually decreases as the BTX-A action is lost. The effect of BTX-A on the external urethral sphincter lasts for only three to 4 months, so patients require repeated injections; this negatively affects patient adherence. In this study, we used BTX-A (Prosigne®^)^ to treat UAB. In clinical terms, Prosigne® can achieve satisfactory results in patients with detrusor overactivity, interstitial cystitis, or UAB. It is also inexpensive.

Unfortunately, the limitation of this study is that the sample size was small and heterogeneous. It is not clear how effective the BTX-A was in the subgroup of patients with neurogenic diseases. In the future, we need to increase the sample size to further clarify which subgroup BTX-A was better for.

## Conclusions

Prosigne® urethral sphincter injection is a highly effective, minimally invasive, and inexpensive method for the treatment of UAB.

## Data Availability

The datasets used and/or analyzed during the current study are available from the corresponding author on reasonable request.

## Abbreviations

BTX-A: Botulinum toxin type A
DLPP: Detrusor leak point pressure
Pura.max: Maximum urethral pressure
Qmax: Maximum urinary flow rate
UAB: Underactive bladder
